# Targeted treatment of solid tumors in pediatric precision oncology

**DOI:** 10.3389/fonc.2023.1176790

**Published:** 2023-05-05

**Authors:** Ilaria Bertacca, Francesco Pegoraro, Annalisa Tondo, Claudio Favre

**Affiliations:** ^1^ Paediatric Hematology/Oncology Department, Meyer Children’s Hospital, Firenze, Italy; ^2^ Department of Health Sciences , University of Firenze, Firenze, Italy

**Keywords:** targeted, childhood, cancer, tumors, precision oncology

## Abstract

The treatment of childhood solid cancer has markedly evolved in recent years following a refined molecular characterization and the introduction of novel targeted drugs. On one hand, larger sequencing studies have revealed a spectrum of mutations in pediatric tumors different from adults. On the other hand, specific mutations or immune dysregulated pathways have been targeted in preclinical and clinical studies, with heterogeneous results. Of note, the development of national platforms for tumor molecular profiling and, in less measure, for targeted treatment, has been essential in the process. However, many of the available molecules have been tested only in relapsed or refractory patients, and have proven poorly effective, at least in monotherapy. Our future approaches should certainly aim at improving the access to molecular characterization, to obtain a deeper picture of the distinctive phenotype of childhood cancer. In parallel, the implementation of access to novel drugs should not only be limited to basket or umbrella studies but also to larger, multi-drug international studies. In this paper we reviewed the molecular features and the main available therapeutic options in pediatric solid cancer, focusing on available targeted drugs and ongoing investigations, aiming at providing a useful tool to navigate the heterogeneity of this promising but complex field.

## Introduction

The treatment of solid and central nervous system (CNS) cancers in children has dramatically evolved in the last decades. The development of intensified cytotoxic chemotherapy and multimodal approaches has led to a significant improvement in survival. In parallel, molecular and diagnostic advances have resulted in more accurate stratification protocols, allowing the selection of those patients who require intensified treatments and reducing long-term toxicities. Nevertheless, pediatric oncologists still have to deal with poorly addressable tumors and severe chemotherapy burdens in cancer survivors (1).

**Figure 1:**
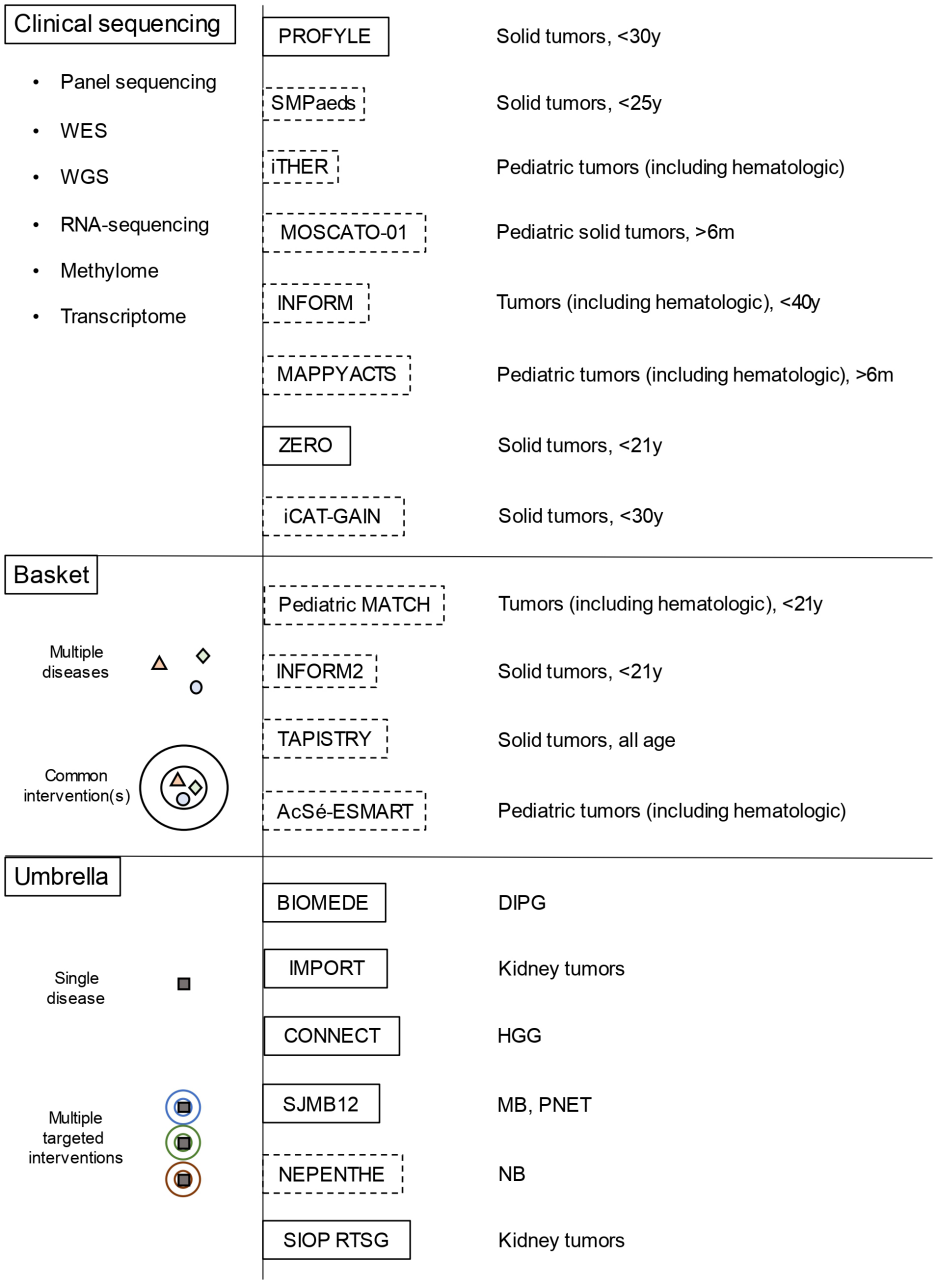
Overview of the main molecular profiling platforms and of basket/umbrella trials for childhood solid cancer (dotted lines represent platforms/trials for relapsed/refractory tumors, while the continuous lines those for newly diagnosed tumors) WES, whole-exome sequencing; WGS, whole-genome sequencing; DIPG, diffuse intrinsic pontine glioma; MB, medulloblastoma; NB, neuroblastoma; HGG, high grade gliomas; PNET, primitive neuroectodermal tumors.

Recently, the implementation of widespread genome-wide profiling programs has contributed to unveiling the genetic heterogeneities and specific nature of childhood solid cancers, as well as their dissimilarity from adult-onset tumors. Moreover, these studies have revealed the contribution of genetic predisposition to pediatric neoplasms and have driven the implementation of targeted approaches (2). Many trials are evaluating pediatric cancer molecular stratification and targeted treatment: the INdividualized therapy FOor Relapsed Malignancies in childhood (INFORM); the individualized THERapy (iTHER) program for children with relapsed or refractory cancer; the MoleculAr Profiling for Pediatric and Young Adult Cancer Treatment Stratification (MAPPYACTS); the pediatric Molecular Analysis for Therapy CHoice (pediatric MATCH); the individualized CAncer Therapy (iCAT) program; the Genomic Assessment Improves Novel Therapy (GAIN) project; the PRecision Oncology For Young peopLE (PROFYLE) program; the Stratified Medicine Paediatrics (SMPaeds) study; and the ZERO childhood cancer program (**Figure 1**). However, among pediatric solid cancers, many entities still lack effective therapeutic strategies, in most cases resulting in poor survival and long-term outcome.

In this review, we will discuss the main genetic abnormalities displayed by pediatric solid tumors, and describe how these abnormalities can be targeted by innovative treatments. Data on already published studies will be provided, as well as available preliminary data on molecules that are still being investigated, with the ultimate goal of providing clinicians with an updated tool for everyday clinical management.

## Neuroblastoma

**Figure 2:**
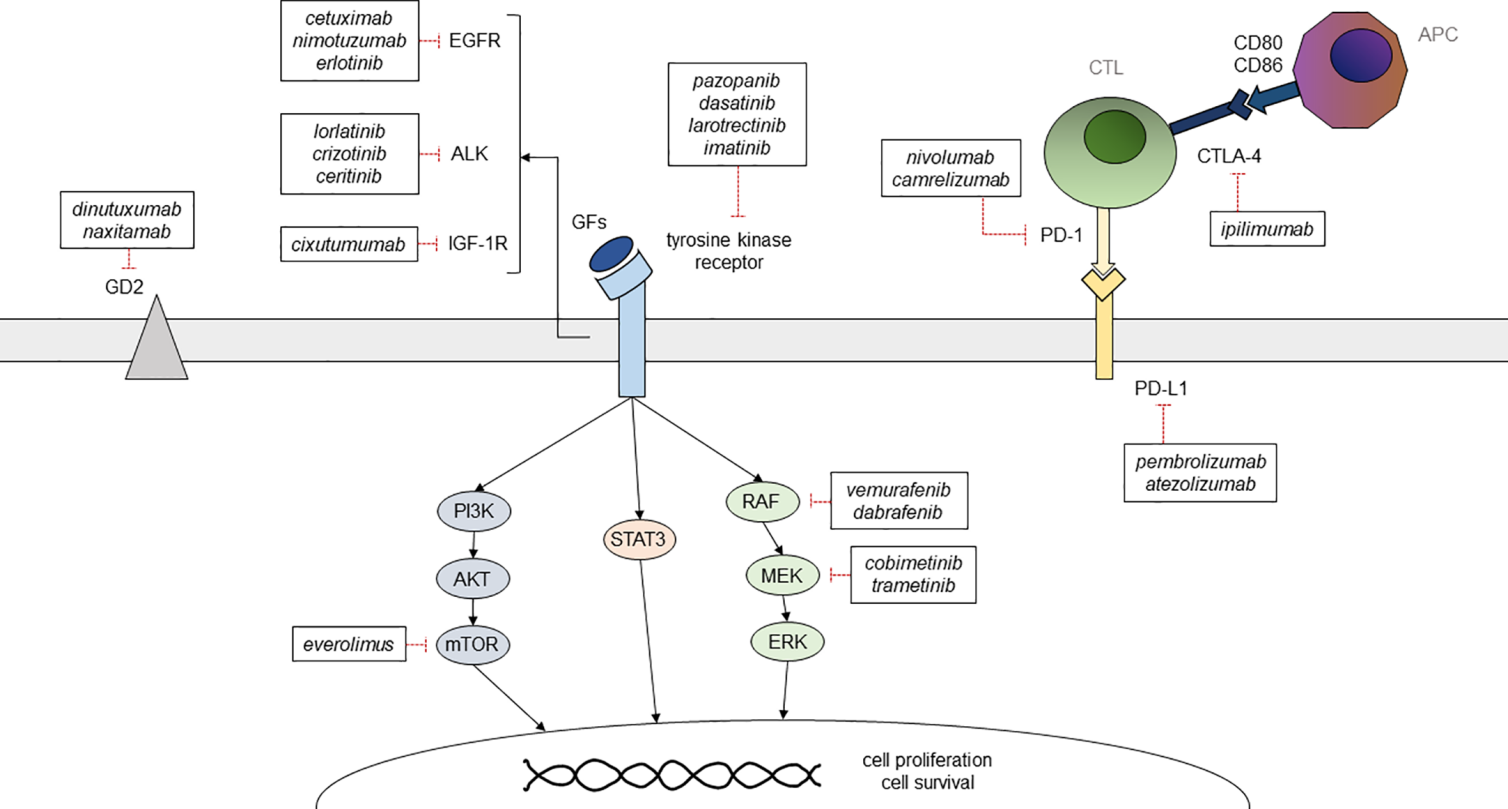
Summary of the main targetable pathways in childhood solid cancer and of the available therapeutic options GFs, growth factors; CTL, cytotoxic T lymphocyte; APC, antigen presenting cell.

Neuroblastoma is a neuroendocrine tumor of the developing sympathetic nervous system and the most common malignancy diagnosed in the first year of life. The most common genetic alterations in neuroblastoma are *MYCN* amplification, anaplastic lymphoma kinase (*ALK*) mutations, segmental chromosomal alterations, and DNA copy number alterations (2, 3). *MYCN* amplification is found in around 20% of cases, typically coexisting with a segmental chromosomal loss of chromosome 1p (4). *ALK* mutations are found in around 10-15% of sporadic neuroblastomas but are also typically responsible for familial forms (5, 6). Rarely, mutations are found in genes of the mitogen-activated protein kinase (MAPK) pathway (e.g., *RAS*, *BRAF*, *PTPN11*, *FGFR*), which are targetable by specific molecules (3) (**Figure 2**). *MYCN* amplification is associated with an aggressive subtype and poor survival, as well as chromosome 11q deletion (7). The activation of telomere maintenance mechanisms (TMMs) by multiple genetic alterations, such as *TERT* rearrangements, *MYCN* amplification, and *ATRX* mutations, is emphasized in relapsed neuroblastoma and has a markedly poor prognosis, especially when associated with MAPK or p53 pathway mutations (8) (**Table 1**).

**Table 1:** Common genetic alterations in pediatric non-CNS solid tumors.

Entity		Molecular alteration
Neuroblastoma		MYCN amplification **ALK** MDM2 or TP53 RAS BRAF PTPN11 FGFR TERT rearrangements ATRX 1p loss 11q deletion
Retinoblastoma		RB1 homozygous deletion MYCN amplification BCOR mut MDM4 amplification
Kidney tumors	Wilms tumors	WT1 TP53 CTNNB1 AMER1 LOH 11p15, 1p, 16q gain of 1q SIX1 or SIX2 MYCN BCOR MAP3K4 BRD7 CREBBP HDAC4 BCORL1 SMARCA4 ARID1A
	Renal cell carcinoma	TFE3 or TFEB rearrangements
	Clear cell sarcoma	BCOR ITDs YWHAE-NUTM2
	Malignant rhabdoid tumor	SMARCB1
	Renal medullary carcinoma	SMARCB1
Sarcomas	Osteosarcoma	TP53 RB1 CDKN CDK4 amplification MDM2 amplification Wnt signaling BMP signaling TGFβ
	Ewing sarcoma	**EWSR1–FLI1** EWSR1–ERG STAG2 TP53 CDKN2A
	Rhabdomyosarcoma	PAX3 or PAX7–FOXO1 LOH of 11p15.5 **RAS** PIK3CA FGFR4 CTNNB1 FBXW7 BCOR TP53 MYOD1 VGLL2 NCOA2 TFPC2 **ALK overexpression**
	Synovial sarcoma	SYT–SSX1 or SSX2
	Alveolar soft part sarcoma	ASPSCR1–TFE3
	Myxoid/round cell liposarcoma	FUS–DDIT3 EWSR1–DDIT3
	Dermatofibrosarcoma protuberans	**COL1A1-PDGFB**
	Infantile fibrosarcoma	**ETV6–NTRK3**
	Undifferentiated sarcomas	CIC or BCOR rearrangements

CNS, central nervous system; LOH, loss of heterozygosity; ITDs, internal tandem duplications

In bold: genetic aberrations that are potential or validated therapeutic targets.

First-line approaches to neuroblastoma consist of chemotherapy, radiotherapy, and autologous hematopoietic stem cell transplantation (HSCT), while the use of anti-GD2 chimeric antibody as maintenance therapy has proven effective in reducing relapse rates (9) (**Figure 2**). First-line chemotherapy combines multiple drugs such as etoposide, vincristine, carboplatin, cisplatin, and cyclophosphamide +/- doxorubicin, depending on the patient’s risk category. Such regimens are followed by myeloablative therapy with busulfan and melphalan in high-risk patients (NCT01728155, NCT01704716). The ALK inhibitor crizotinib has already been used with variable efficacy in pediatric solid cancer, including neuroblastoma (10) (**Figure 2**). At the moment, a phase-III trial comparing iobenguane I-131 meta-iodobenzylguanidine (MIBG) or crizotinib plus standard therapy in high-risk neuroblastoma is ongoing (NCT03126916). Crizotinib is also being evaluated in association with chemotherapy for relapsed neuroblastoma (NCT01606878), while it did not reach adequate response rates in monotherapy (11). Phase-I trials are ongoing using other ALK inhibitors, such as lorlatinib and ceritinib (NCT03107988, NCT01742286) (**Table 2**). Immune checkpoint inhibitors, such as the anti-PD-1 nivolumab, did not prove effective in monotherapy for relapsed neuroblastoma (35, 36), but their association with both targeted molecules and conventional therapies is being tested in newly diagnosed and relapsed neuroblastoma. Also, trials on chimeric antigen receptor T-cell (CAR-T) based therapies targeting CD171 (NCT02311621) and GD2 (NCT02765243) on relapsed neuroblastoma are ongoing (**Table 3**). Nowadays, children with neuroblastoma can also benefit from precision diagnostic and therapeutic trials such as the PEDS-PLAN (NCT02559778) and the NEPENTHE (NCT02780128) studies (**Figure 1**).

**Table 2:** Targeted therapies towards specific mutations.

Target	Drug	Indication	Reference
NTRK fusion	Larotrectinib Entrectinib	Soft-tissue sarcomas Glioma	(12, 13)
ALK	Crizotinib Lorlatinib Ceritinib	Neuroblastoma Rhabdomyosarcoma	(10, 14) NCT03126916 NCT01606878 NCT03107988 NCT01742286
EGFR	Cetuximab Nimotuzumab Erlotinib CAR-T	Retinoblastoma Wilms tumor Glioma	NCT03618381 NCT03638167
EZH2	Tazemetostat	Malignant rhabdoid tumor Soft-tissue sarcomas ATRT	NCT02601937
Multi-TKI	Cabozantinib Regorafenib Pazopanib Sorafenib Imatinib Sunitinib Avapritinib Dasatinib	Renal cell carcinoma Osteosarcoma Ewing sarcoma Soft tissue sarcomas CNS germ cell tumors	(15–22) NCT02389244 NCT02048371 NCT04773782
PI3K/AKT/mTOR pathway	Everolimus Temsirolimus Perifosine	Osteosarcoma Ewing sarcoma Rhabdomyosarcoma Glioma Ependymoma ATRT	(23–29) NCT01222715 NCT01734512 NCT04485559 NCT02155920 NCT02574728
EWSR1-FL1 - RNA Helicase A	YK-4-279 TK216	Ewing sarcoma	NCT02657005
HRAS	Tipifarnib	Rhabdomyosarcoma	NCT04284774
BRAF	Dabrafenib Tovorafenib	Glioma	(30) NCT02684058 NCT04775485
MEK	Trametinib Selumetinib Cobimetinib	Glioma	(30–34) NCT02684058

TKI, tyrosine kinase inhibitors; CAR-T, Chimeric Antigen Receptor T cell therapies; CNS, central nervous system; ATRT, atypical teratoid/rhabdoid tumor.

**Table 3:** Immune-based therapies.

Target	Drug	Indication	Reference
GD2	Dinutuximab Naxitamab CAR-T	Neuroblastoma Ewing sarcoma Glioma	(9, 37) NCT02765243 NCT04196413 NCT04099797
CD171	CAR-T	Neuroblastoma	NCT02311621
PD-1	Nivolumab Camrelizumab CAR-T	Neuroblastoma Renal cell carcinoma Osteosarcoma Soft-tissue sarcomas ATRT	(38, 39) NCT04433221 NCT05407441
PD-L1	Pembrolizumab Atezolizumab	Soft-tissue sarcomas Glioma Ependymoma Medulloblastoma ATRT	(40) NCT02359565 NCT05286801
CTLA-4	Ipilimumab	Renal cell carcinoma Osteosarcoma Rhabdomyosarcoma Soft-tissue sarcomas ATRT	(38) NCT05407441
B7-H3	Enoblituzumab CAR-T	Retinoblastoma Wilms tumor Ewing sarcoma HGG	NCT04483778 NCT02982941 NCT04185038 NCT04897321
NOD2	Mifamurtide	Osteosarcoma	(41)
TIGIT	Tiragolumab	ATRT	NCT05286801

CAR-T, Chimeric Antigen Receptor T cell therapies; CNS, central nervous system; HGG, high grade glioma; ATRT, atypical teratoid/rhabdoid tumor; TIGIT, T cell immunoreceptor with Ig and ITIM domains.

## Retinoblastoma

Retinoblastoma is a rare tumor of retinal progenitor cells that accounts for around 2-3% of childhood cancer (42). Biallelic mutations inactivating *RB1* are the most common drivers of both sporadic and familial retinoblastoma, but also *MYCN* amplification and *BCOR* mutation can be involved (43); less frequently, copy number alterations (e.g., involving *MDM4*) are found (44) (**Table 1**).

The treatment of retinoblastoma relies on a multimodal approach based on risk stratification (depending on disease staging, extraocular involvement, and germline mutations) and available institutional resources (45). Adjuvant intravenous chemotherapy with vincristine, etoposide, and carboplatin can be administered in patients with high-risk features (46), and new approaches include intra-arterial delivery of chemotherapy agents such as melphalan, topotecan, or carboplatin (47). However, despite the thorough molecular profiling of retinoblastoma, targeted approaches have not been investigated, except for CAR-T-based therapies directed against EGFR806 (NCT03618381) or B7H3 (NCT04483778) in the context of large-spectrum phase-I trials (**Tables 2**, **3**).

## Kidney tumors

Wilms tumor (WT), also known as nephroblastoma, is the most common kidney tumor in childhood (90%). Less common pediatric kidney cancers include renal cell carcinoma (RCC) (5%), clear-cell sarcoma of the kidney (CCSK) (3.5%), congenital mesoblastic nephroma (4%), malignant rhabdoid tumor (MRT) (1.5%), and other rare cancers such as cystic nephroma and metanephric tumors (2%) (48).

### Wilms tumor

Aberrations of *WT1* and *TP53*, Wnt pathway activating mutations involving *CTNNB1* and *AMER1*, and loss of heterozygosity (LOH) of 11p15 resulting in overexpression of IGF2 are known to be associated with WT. Abnormalities of 11p15 methylation, as well as 1q gain, LOH of 1p, and 16q were shown to be prognostic biomarkers for inferior survival (49). Recently, whole-exome sequencing analyses identified novel mutations involving microRNA processing genes, renal developmental genes *SIX-1* and *SIX-2*, *MYCN*, histone modification mediators such as *BCOR*, *MAP3K4*, *BRD7*, *CREBBP*, and *HDAC4*, transcriptional repressors such as *BCORL1* and epigenetic remodelers *SMARCA4* and *ARID1A* (50, 51) (**Table 1**).

Standard treatment of WT includes surgery, pre- and post-operative chemotherapy, and radiation in advanced-stage disease and in intermediate or high-risk histology. According to the International Society of Paediatric Oncology (SIOP) protocol, first-line chemotherapy consists of a combination of vincristine, dactinomycin, and doxorubicin; etoposide and cyclophosphamide are used in the Children’s Oncology Group (COG) protocol (52). Several molecules, such as the IGF-1R inhibitor cixutumumab, the multi-tyrosine kinase inhibitors (TKIs) sorafenib and cabozantinib, and the aurora A kinase inhibitor alisertib, were used in children and young adults with refractory solid tumors including WT, but no relevant clinical activity was demonstrated (53–55). A phase I trial is ongoing to evaluate the efficacy of vorinostat, a histone deacetylase inhibitor, in combination with standard chemotherapy in patients with recurrent and refractory solid tumors such as WT (NCT04308330). Also, the combination of antibodies directed against tumor cells antigens and an anti-cancer drug is being studied in WT. A phase II trial of the anti-CD56 antibody lorvotuzumab linked with the anti-mitotic agent mertansine showed good tolerability, but results on efficacy are still expected (NCT02452554) (**Table 4**). Recently, some interest has been addressed regarding B7-H3 (CD276), a checkpoint molecule that was found to be overexpressed in WT and probably related to unfavorable prognosis; promising preclinical data on the anti-B7-H3 antibody-drug conjugate are available in xenografts models of different pediatric solid tumors including WT (60). Moreover, enoblituzumab, a monoclonal antibody directed against CD276 in children with B7-H3-expressing WT (NCT02982941), and CAR-T cell immunotherapy targeting B7-H3 (NCT04483778) and EGFR (NCT03618381) are being tested in WT (**Tables 2**, **3**).

**Table 4:** Other targeted therapies for pediatric solid tumors.

Target	Drug	Indication	Reference
AAK	Alisertib	Malignant rhabdoid tumor ATRT	NCT02114229
HDAC	Vorinostat Entinostat	Wilms tumor Rhabdomyosarcoma	NCT04308330 NCT02780804
CD56	Lorvotuzumab mertansine	Wilms tumor Rhabdomyosarcoma	NCT02452554
RANKL	Denosumab	Osteosarcoma	NCT02470091
HER-2	Trastuzumab CAR-T	Osteosarcoma Glioma Ependymoma	NCT04616560 NCT04433221 NCT03500991 NCT02442297 NCT04903080
IGF-1R	Cixutumumab CAR-T	Ewing sarcoma	(24, 37)
PARP	Talazoparib	Ewing sarcoma	(56)
BRD4	JQ1	Rhabdomyosarcoma	(57)
JAK1	Itacitinib	Soft tissue sarcomas	NCT03670069
VEGF	Bevacizumab	Rhabdomyosarcoma Soft tissue sarcomas Glioma Ependymoma Embryonal tumors	(33, 34) NCT01356290
IL13Rα2	CAR-T	Glioma	NCT02208362
CDK4/6	Ribociclib	Ependymoma Embryonal tumors	(29)
SHH	Vismodegib Sonidegib	Medulloblastoma	(58, 59)
CHK1/2	Prexasertib Silmitasertib	Medulloblastoma	NCT04023669 NCT03904862

CAR-T, Chimeric Antigen Receptor T cell therapies; ATRT, atypical teratoid/rhabdoid tumor.

### Other kidney tumors

RCC in children and adolescents is characterized by translocations involving the *TFE3* gene, located on chromosome X, or, less frequently, the *TFEB* gene. *TFE3* fusion partners include *ASPL*, *PRCC*, *SFPQ*, and others. The *TFEB* gene is most commonly fused with the *MALAT1* gene (61) (**Table 1**). RCC treatment consists of surgery and adjuvant therapy. TKIs such as sunitinib and axitinib have been tested in pediatric RCC with promising results (15) (**Table 2**). The combination of TKIs with immune-checkpoint inhibitors (CPIs) (anti-CTLA-4, anti-PD-1/PD-L1) achieved higher response rates, and it is now recommended as first-line therapy in adults with metastatic RCC. In childhood, CPIs have only been evaluated in early-phase trials showing safety, tolerability, and variable clinical efficacy (38) (**Table 3**).

CCSK is the third most common pediatric kidney tumor. Internal tandem duplications (ITDs) in the *BCOR* gene are the prevalent genetic aberrations (70%) in this entity and are mutually exclusive with the less common chromosomal translocation t(10;17), which results in the *YWHAE*-*NUTM2* gene fusion (62). So far, no targeted therapies directed against these molecular features have been developed. MRT is primarily driven by the loss of the *SMARCB1* gene (63) (**Table 1**). Phase I/II studies on the EZH2 inhibitor tazemetostat in children with SMARCB1-deficient solid tumors are underway with promising results (NCT02601937) (**Table 2**), and preclinical trials are evaluating other potential therapeutic agents, such as aurora A kinase inhibitors, MDM2/4 inhibitors and proteasome inhibitors (48). Renal Medullary Carcinoma (RMC), a non-clear-cell RCC, has been associated with *SMARCB1* deficiency (**Table 1**). Novel therapies effective against MRT may also be useful for this subgroup of RCC.

## Sarcomas

Sarcomas are a group of solid tumors developing from mesenchymal cells that can affect bone and soft tissues. Each subtype has a different phenotype and distinct genetic features.

### Osteosarcoma

Osteosarcoma is the most common primary malignant bone tumor occurring in children and adolescents. It is characterized by a high level of genomic instability, probably consequent to mutations in genes that are essential for mitotic checkpoints, such as the inactivation of *TP53* and the *RB1* tumor suppressor genes. Less frequently, loss of *CDKN* genes and amplification of *CDK4* have been reported. In some cases, p53 inactivation indirectly results from *MDM2* amplification (64) (**Table 1**). Gain-of-function mutations in the effectors of PI3K/Akt pathway can be found in a high percentage of osteosarcomas, especially in advanced stages (65). Aberrant expression of genes involved in bone cell differentiation, such as Wnt family and BMP/TGFβ family members, has also been associated with osteosarcomagenesis (66). There is no evidence of reliable molecular prognostic factors for osteosarcoma, but the expression of P‐glycoprotein (Pgp), an efflux pump that removes chemotherapeutic drugs from cells, has been associated with poorer survival in patients affected by osteosarcoma (41).

The standard treatment of osteosarcoma consists of a combination of chemotherapy and surgery, with a poor prognosis for patients with metastatic (usually to the lung) or relapsed disease (66). Methotrexate, doxorubicin, and cisplatin represent the backbone of the medical treatment, and poor responders also receive high-dose ifosfamide (41). In recent years, TKIs have had an increasing role in the treatment of osteosarcoma: cabozantinib in patients with advanced or recurrent osteosarcoma and Ewing sarcoma (16); anlotinib in unresectable or metastatic bone sarcomas (17); regorafenib in recurrent, progressive and metastatic bone sarcomas (NCT02389244); and pazopanib in recurrent osteosarcoma metastatic to the lung (18) (**Table 2**) (**Figure 2**). A phase II randomized study is still ongoing to evaluate the efficacy and safety of lenvatinib in combination with chemotherapy in relapsed and refractory osteosarcoma (NCT04154189). mTOR inhibitors have been used in osteosarcomas, showing poor antineoplastic activity as monotherapy (67), probably due to the presence of many feedback loops in the IGF/PI3K/mTOR pathway. Combination strategies co-targeting two or more proteins are being evaluated in order to avoid drug resistance. The multitargeted TKI sorafenib in combination with the mTOR inhibitor everolimus (**Figure 2**) proved to be effective in unresectable osteosarcoma progressing after standard treatment, but it did not reach the prespecified target of 6-month progression-free survival (PFS) of 50% (23) (**Table 2**). Similarly, immune checkpoint inhibitors such as anti-PD-1 and anti-CTLA-4 antibodies have been investigated with limited activity when used as a single agent (NCT02406781; ADVL1412) (68), but the combination of the anti-VEGFR apatinib and the PD-1 inhibitor camrelizumab seemed to prolong PFS in comparison to apatinib alone in advanced osteosarcoma but did not achieve the prespecified target of 6-month PFS of 60% (39). Concerning immunotherapies, the Italian Sarcoma Group led a phase II trial showing the benefit of adjuvant mifamurtide – an immune-stimulating compound that promotes macrophage and monocyte antitumor activity – in patients with non-metastatic osteosarcoma expressing P-glycoprotein (Pgp+) (41) (**Table 3**). Denosumab, a monoclonal antibody directed against RANKL – of which overexpression has been related to poorer outcomes – is being investigated in patients with recurrent or refractory osteosarcoma (NCT02470091). Another phase II trial is evaluating the efficacy of the anti-HER-2 monoclonal antibody trastuzumab linked to the topoisomerase-I inhibitor deruxtecan (NCT04616560) (**Table 4**). Finally, innovative CAR-T cell based approaches are being evaluated in multiple sarcomas (NCT04433221) (**Tables 3**, **4**). Potential targets include surface antigens overexpressed by osteosarcoma cell lines, such as HER-2 and PD-1.

### Ewing sarcoma

Ewing sarcoma (EWS) is the second most common pediatric malignant bone tumor, but it can also occur in soft tissues. The translocation between the *EWSR1* gene and the *FLI1* gene is the most common (85%), resulting in a fusion product that functions as an oncoprotein. Less frequently, the translocation of *EWSR1* involves other members of ETS family transcription factors, such as *ERG* (10%), or non-ETS family genes, such as *NFACT2*. A few additional pathogenic alterations have been observed, like loss-of-function mutations involving *STAG2*, *TP53*, and *CDKN2A* genes (69, 70) (**Table 1**). STAG2 and *TP53* mutations have been associated with a dismal prognosis, especially when coexisting, as for the loss of *CDKN2A* (69). Rearrangement of the *CIC* and *BCOR* genes have been implicated in some cases of small round cell sarcomas, also defined as “Ewing-like” because of their clinical and morphological similarities with EWS, but sometimes with a worse prognosis due to a poorer response to treatments (71, 72).

Standard treatment for Ewing sarcoma relies on combined chemotherapy (*e.g.*, vincristine, doxorubicin, ifosfamide, cyclophosphamide, etoposide, and dactinomycin), radiotherapy, and surgery approach, less frequently including autologous HSCT in cases requiring high-dose chemotherapy with busulfan and melphalan (73). Targeting the EWSR1-FLI1 fusion protein is not easy, due to its structure and the lack of enzymatic activity. However, molecules inhibiting the interaction between EWSR1-FLI1 and the RNA Helicase A might be effective: YK-4-279 has shown promising results in preclinical studies (74, 75), and a phase I clinical trial is ongoing to evaluate the efficacy of TK216 in combination with vincristine (NCT02657005) (**Table 2**). IGF-1R targeted antibodies have been evaluated in advanced-stage EWS, inducing a short-term response when used as a single agent, but an improvement of PFS when combined with mTOR inhibitors (cixutumumab/temsirolimus) (24, 76). A phase I trial of the PARP inhibitor talazoparib in combination with irinotecan +/- temozolomide showed promising results in recurrent and refractory solid tumors, including EWS (56) (**Table 4**). The multitargeted TKI cabozantinib demonstrated antitumor activity in patients with advanced or recurrent EWS and osteosarcoma (16), while a phase II trial using regorafenib is still ongoing with promising early results (NCT02048371) (**Table 2**). Finally, CAR-T cell based therapy is currently being investigated in preclinical trials using *in vitro* and *in vivo* xenograft models, showing promising antitumor activity. Potential targets include VEGFR2, IGF1R, ROR1, GD2, B7-H3, EphA2, and NKG2D (37) (**Tables 3**, **4**).

### Rhabdomyosarcoma

Rhabdomyosarcoma (RMS) is the most common soft-tissue sarcoma in childhood and adolescence. It is classified on the basis of genetic and morphologic features into embryonal, alveolar, spindle cell, and pleomorphic RMS (77). Alveolar RMS (ARMS) is the second most common subtype (20%) and it is usually characterized by the translocation between *PAX* and *FOXO1* genes: *PAX3-FOXO1* is the most common (75%), while *PAX7-FOXO1* occurs in 10% of cases. The alveolar histologic subtype is an unfavorable prognostic factor that classifies the patient within the very high-risk group. Embryonal rhabdomyosarcoma (ERMS), the most frequent subtype (70-80%), has a wider range of genetic aberrations and a higher mutation burden compared to ARMS. The most common chromosomal aberration – in up to 50% of cases – is the loss of heterozygosity at 11p15.5. Various mutations involve the RTK/RAS/PIK3CA pathway, including *RAS* (approximately 25% of cases of fusion-negative RMS), *PIK3CA*, and *FGFR4*. Also, cell cycle regulatory genes and tumor suppressors were found to be altered, including *CTNNB1*, *FBXW7*, *BCOR* and *TP53* (78). Spindle cell RMS (ssRMS) often harbors *MYOD1* mutation, which is associated with poor prognosis (79). Other recurrent aberrations include gene fusions involving *VGLL2*, *NCOA2*, and *TFPC2* (80, 81). Furthermore, ALK overexpression has been reported, especially in ARMS (82) (**Table 1**).

The standard treatment of RMS includes surgery, chemotherapy, and radiation therapy (83). The first-line chemotherapy regimens often include vincristine, dactinomycin, and ifosfamide, with or without doxorubicin. In high-risk groups, maintenance chemotherapy consisting of vinorelbine and cyclophosphamide showed an improvement in overall survival (84). Therapies that directly inhibit PAX-FOXO1 fusion protein with good specificity and affinity are yet to be designed. A promising strategy is to target *BRD4*, an epigenetic reader that mediates *PAX-FOXO1* transcription through the novel molecule JQ1, which reduces the expression of the oncogenic fusion protein (57) (**Table 4**). Various receptor tyrosine kinase (RTK) inhibitors have been tested in RMS. An ongoing phase II trial is evaluating the efficacy of tipifarnib, an indirect HRAS inhibitor, in pediatric patients with advanced or recurrent *HRAS* mutated solid tumors (NCT04284774). The mTOR inhibitor temsirolimus has been tested in relapsed RMS with a satisfying antitumor response, achieving superior event-free survival rates compared with bevacizumab (NCT01222715). A phase II clinical trial is ongoing exploring the ALK inhibitor crizotinib in patients with advanced tumors induced by causal alterations of either *ALK* or *MET* (2011-001988-52), while there are promising preclinical data on the ALK inhibitor ceritinib combined with dasatinib, an Src family kinase inhibitor (14) (**Table 2**). Conversely, no sustained response has been achieved by targeting IGF-1R. The addition of the anti-IGF-1R cixutumumab to multiagent chemotherapy for metastatic RMS did not improve survival (85), while a phase II trial studying the efficacy of the IGF-1R monoclonal antibody ganitumab in combination with dasatinib in relapsed and refractory RMS (NCT03041701) was closed early due to lack of the study drug. As discussed for WT, since CD56 is expressed on several tumors cells, the antibody-drug conjugate lorvotuzumab mertansine was evaluated in recurrent solid cancers including RMS but results on efficacy are pending (NCT02452554), as is for entinostat, an oral histone deacetylase inhibitor that has been evaluated in a phase I trial in pediatric patients with recurrent or refractory solid tumors (NCT02780804) (**Table 4**).

### Non-rhabdomyosarcoma soft tissue sarcomas

The non-rhabdomyosarcoma soft tissue sarcoma (NRSTS) group includes multiple histological variants, and pathognomonic chromosomal aberrations have been identified in certain subtypes. Synovial sarcoma has been associated with *SYT-SSX1/2* translocation (86). Alveolar soft part sarcoma (ASPS) often carries a translocation between *ASPSCR1* and *TFE3* genes, resulting in a fusion protein that transcriptionally upregulates *MET* (87). Myxoid/Round cell Liposarcoma (MRLPS) represents 20-30% of LPS and it is the only subtype described in childhood and adolescence. Round cell LPS is defined as having more than 5% of small round cells in a myxoid LPS. Most MRLPSs carry a pathognomonic translocation between the *FUS* gene and the *DDIT3* gene (also known as *CHOP*), whereas a smaller proportion is associated with *EWSR1-DDIT3* translocation. Overexpression of p53 in myxoid LPS has been associated with poor prognosis (88). Dermatofibrosarcoma protuberans is characterized by a *COL1A1-PDGFβ* translocation in up to 90% of cases, resulting in autocrine stimulation of the PDGF receptor (89). The *ETV6-NTRK3* gene fusion is pathognomonic for infantile fibrosarcoma (70-100% of cases) (90) (**Table 1**).

Surgery remains the mainstay of treatment for NRSTS, while radiation and chemotherapy with doxorubicin and ifosfamide can be administered as a neoadjuvant or adjuvant treatment to improve the efficacy of surgery or in patients deemed at high risk for metastasis (91). Although multi-TKI pazopanib (**Figure 2**) is not approved for many STSs such as LPS, RMS apart from alveolar and pleomorphic subtypes, and dermatofibrosarcoma protuberans, it is occasionally used off-label based on published studies on its antitumor activity (19). Other TKIs such as sunitinib and cediranib also achieved tumor responses or disease stabilization in ASPS (20) (**Table 2**). Unlike for other NRSTSs, the anti-PD-L1 antibody pembrolizumab (**Figure 2**) has shown some efficacy in ASPS. Indeed, the combination of immune checkpoint inhibitors with anti-angiogenic therapies has achieved significant improvements in response rates (40) (**Table 3**). Similarly, the TRK inhibitor larotrectinib (**Figure 2**) showed encouraging antitumor activity in pediatric patients with TRK fusion-positive tumors, including STSs (12). Moreover, the PDGFR inhibitor imatinib mesylate (**Figure 2**) proved to be effective in recurrent, unresectable, and metastatic dermatofibrosarcoma protuberans, mainly in patients with a t(17;22) translocation (21). Also, interim results from a phase I study of the EZH2 inhibitor tazemetostat in relapsed or refractory INI1-negative tumors (e.g., epithelioid sarcoma, extraskeletal myxoid chondrosarcoma, dedifferentiated chordoma) or synovial sarcoma showed promising anti-tumor activity (NCT02601937) (**Table 2**). An ongoing phase I trial is evaluating the efficacy of the JAK1-selective inhibitor itacitinib in patients with refractory advanced or metastatic sarcomas (NCT03670069) (**Table 4**). Finally, a modified T cell receptor (TCR) based immunotherapy directed against NY-ESO-1, which is expressed in 90% of MRLPS tumors, has shown promising preliminary results in adults (NCT02992743).

## Central nervous system

As a group, central nervous system (CNS) tumors are the most common solid neoplasm during childhood and the leading cause of cancer-related mortality in this age group. Among CNS tumors, gliomas account for approximately 50% of cases in children aged 0-14 years (92); they include several histological variants such as low and high-grade gliomas, other astrocytomas, ependymomas, and oligodendrogliomas.

### Low-grade gliomas

Low-grade gliomas (LGGs) are the most common pediatric brain tumors. LGGs usually occur sporadically, but they can be associated with cancer-predisposition syndromes such as Neurofibromatosis type 1 (NF1) and Tuberous Sclerosis (TS) (93). The presence of NF1 has been reported as a favorable prognostic factor in optic pathway glioma (33). LGGs often carry *BRAF* gene fusions (*e.g.*, *KIAA1549-BRAF*) or activating mutations (*e.g.*, *BRAF V600E*), *NF1* mutations, *RAF* fusions, *FGFR1* mutation or rearrangement, impacting both the RAS/MAPK and PI3K/AKT/mTOR pathways. The *BRAF V600E* mutation seems to correlate with a poorer prognosis across a broad spectrum of pediatric LGG (34). Gangliogliomas, a subset of glioneuronal tumors, often harbor the activating *BRAF V600E* mutation as well (94). Rearrangements of MYB or MYBL1 occur most frequently in diffuse LGGs (95, 96) (**Table 5**).

**Table 5:** Common genetic alterations in pediatric CNS solid tumors.

Entity		Molecular alteration
Low-grade gliomas		KIAA1549-BRAF **BRAFV600E** **NF1** RAF fusion FGFR1 mut or rearrangement MYB or MYBL1 rearrangements
High grade glioma	Diffuse midline glioma H3K27 altered	**K27M** EZHIP overexpression EGFR TP53 ATRX
	Diffuse hemispheric glioma H3G34 mutant	H3F3A **PDGFRA amplification** CCND2 amplification TP53 ATRX
	Diffuse high grade glioma H3 and IDH wildtype	TP53 MYCN or EGFR amplifications PDGFRA mut or amplification
	Infant type hemispheric glioma	**NTRK fusions** ROS1, ALK or MET fusions
Ependymoma		YAP1 fusions ZFTA fusions Loss of H3K27 trimethylation EZHIP mut or overexpression MYCN amplification
Medulloblastoma	WNT-activated MB	CTNNB1 ch 6 monosomy APC DDX3X SMARCA4 TP53 CSNK2B PIK3CA EPHA7
	SHH-activated MB	TP53 PTCH1 SUFU SMO MYC, MYCN, GLI1/2 amplifications losses of 9q, 10q, 14q, 17p gains of 2, 3q, 9p
	Group 3	MYC/MYCN and OTX2 amplifications isochromosome 17q SMARC4 KBTBD4 CTDNEP1 losses of 8, 10q, 11 and 16q gains of 1q, 7 and 18
	Group 4	MYC/MYCN, OTX2, CDK6 amplifications isochromosome 17q PRDM6 overexpression KDM6A ZMYM3 KTM2C KBTBD4 losses of 8, 11p and X gains of 7 and 18
ATRT		**SMARCB1** **SMARCA4** SHH, Notch, Melanosomal pathway, MYC and Hox cluster overexpression
Germ cell tumors		**KIT** RAS CBL AKT1 gains of 12p, X loss of 13q
Others		FOXR2 rearrangements BCOR ITDs DICER1 CIC rearrangements MN1 rearrangements

CNS, central nervous system; MB, medulloblastoma; ATRT, atypical teratoid/rhabdoid tumor; ITDs, internal tandem duplications.

In bold: genetic aberrations that are potential or validated therapeutic targets.

The mainstay of LGGs treatment is complete surgical resection. When radical surgery is not feasible, chemotherapy or radiotherapy may be used to treat the residual lesions. The standard chemotherapy regimens recommended for pLGGs include carboplatin and vincristine, or vinblastine monotherapy (97). Recently, the Food and Drug Administration (FDA) approved the use of the BRAF inhibitor dabrafenib in combination with the MEK inhibitor trametinib (**Figure 2**) for pediatric patients with BRAF V600E mutated LGG, based on a large phase II open label trial (NCT02684058). A phase II multicentre trial showed some efficacy of the MEK inhibitor selumetinib in recurrent, refractory, and progressive pLGG carrying BRAF aberrations and NF1 mutations (31). Cobimetinib, another MEK inhibitor (**Figure 2**), proved to be safe and effective in LGGs with MAPK pathway activation (32). Recently, the pan-RAF inhibitor tovorafenib (DAY101), provided encouraging response data in pediatric and young adult pretreated patients with recurrent or progressive low-grade glioma or advanced solid tumors harboring a known activating *BRAF* alteration (NCT04775485). The selective mTOR inhibitor everolimus (**Figure 2**) is approved for TS-associated subependymal giant cell astrocytoma and is also tolerable and effective in terms of disease stabilization in sporadic pLGGs (NCT01734512) (25) (**Table 2**). Finally, the treatment with bevacizumab, an antibody directed against VEGF, has shown good short-term disease control, even if several patients progressed after the drug discontinuation (98, 99) (**Table 4**).

### High-grade gliomas

High-grade gliomas (HGGs) are grade III-IV tumors that still have a very poor prognosis. According to the 2021 World Health Organization (WHO) classification of CNS tumors, pediatric diffuse high-grade gliomas include several categories of which the two major entities are diffuse midline glioma (DMG) H3K27 altered and diffuse hemispheric glioma H3G34 mutant (100).

Concerning the first subgroup, the loss of H3K27 trimethylation can result from *K27M* mutations, EZHIP overexpression, or *EGFR* mutations (101). Of note, *H3K27* mutations have been associated with poor prognosis (102). Of note, the DMG entity now includes diffuse intrinsic pontine gliomas in addition to diffuse gliomas arising in other midline locations. The second main entity, diffuse hemispheric glioma, is typically characterized by a missense mutation in the *H3F3A* gene. *PDGFRA* and *CCND2* amplifications are less common and are both associated with poor outcomes. *TP53* and *ATRX* mutations are detected in almost all cases of diffuse hemispheric glioma H3G34 mutant, but they can also occur in *H3K27*-mutated diffuse midline gliomas (103). The H3- and IDH-wildtype diffuse HGG represents an additional entity, which by definition lacks alterations in histone *H3*, *IDH1*, and *IDH2* genes. This group exhibits great molecular heterogeneity, including *TP53* mutations, *MYCN* and *EGFR* amplifications, and *PDGFRA* mutation or amplification (103). *MYCN*-mutated high-grade gliomas are associated with a poor prognosis. Finally, the infant-type hemispheric glioma category often carries RTK fusions involving the *NTRK*, *ROS1*, *ALK*, and MET genes, while BRAF V600E mutations are observed in 10-15% of pediatric HGGs (102, 103) (**Table 5**).

The standard treatment of HGG Is based on surgical resection followed by radiation therapy +/- concurrent chemotherapy (104). Temozolomide is the most commonly used conventional drug for newly diagnosed pHGGs (102). Many TKIs have been investigated in HGGs but in most cases did not provide sufficient disease control when investigated in monotherapy (105, 106). However, the multi-TKI entrectinib showed durable responses in children with solid tumors with *NTRK1/2/3* or *ROS1* fusions, including primary brain tumors (13). A phase I/II multicenter trial of avapritinib in pediatric relapsed and refractory solid tumors harboring mutations in *KIT* or *PDGFRA* and *H3K27* altered gliomas is recruiting (NCT04773782). Moreover, preliminary results of recent ongoing trials suggest promising results when TKIs are used in combination with other targeted therapies, such as the mTOR inhibitor everolimus (26) (NCT04485559). In addition, a phase I study demonstrated the feasibility of the combination therapy with temsirolimus and the AKT inhibitor perifosine in recurrent and refractory pediatric solid tumors, including HGGs (27) (**Table 2**). On the other hand, the EGFR inhibitors cetuximab and nimotuzumab (**Figure 2**) were evaluated in addition to standard treatment in pediatric HGGs, but both were not able to markedly improve overall survival compared to controls (107, 108). A phase II trial has evaluated the use of the BRAF inhibitor dabrafenib in combination with the MEK inhibitor trametinib (**Figure 2**) in children and adolescents with relapsed and refractory BRAF-mutated high-grade glioma (NCT02684058). The efficacy and safety of this combination therapy in these patients have already been reported in some case series (30) (**Table 2**). Finally, multiple trials are evaluating CAR-T cell immunotherapy targeting B7-H3-expressing pontine DMG (NCT04185038) or solid tumors (NCT04897321), HER-2 in recurrent or refractory HER2-positive CNS tumors (NCT03500991, NCT02442297), IL13Rα2 in recurrent or refractory malignant gliomas (NCT02208362), EGFR in pediatric recurrent or refractory EGFR positive CNS tumors (NCT03638167), and GD2-expressing CNS tumors including DMG (NCT04196413, NCT04099797) (**Tables 2**–**4**).

### Ependymoma

Based on its anatomic location, ependymoma can be classified into three main groups: supratentorial, posterior fossa, and spinal cord (109). Each of these groups consists of different clinical, genetic, and histopathological subtypes. Supratentorial ependymomas generally have a more favorable prognosis. According to the 2021 WHO classification of CNS tumors, there are two main subsets of supratentorial ependymoma, namely the ZFTA (also called C11orf95) and the YAP1 fusion positive supratentorial ependymomas. Regarding the first entity, the ZFTA-RELA fusion protein is the most frequently identified alteration. Those that have neither the *ZFTA* nor the *YAP1* fusion constitute a different supratentorial ependymoma subset (100). Posterior fossa ependymomas are distinguished into PFA (pediatric type) and PFB, which mainly occur in older children and in adults, and have a better prognosis. Posterior fossa ependymomas lack recurrent mutations. However, PFA often exhibits loss of *H3K27* trimethylation and overexpression of *EZHIP*. Among spinal cord ependymomas, *MYCN* amplification defines a distinctive subtype with poorer outcomes (109, 110) (**Table 5**).

Surgical resection followed by radiation therapy to the tumor bed is the pivotal treatment of ependymomas and often guarantees a good long-term prognosis. Current treatment approaches do not include chemotherapy in most cases. However, there are few treatment options for recurrent disease besides re-irradiation (111). Only a few targeted therapies have been investigated in ependymoma. The role of antiangiogenic agents in pediatric brain tumors remains controversial, due to conflicting results (112, 113). A phase II study, aiming to evaluate the efficacy of the anti-VEGF antibody bevacizumab in children with recurrent and progressive medulloblastoma, ependymoma, or the atypical teratoid rhabdoid tumor (ATRT) is recruiting (NCT01356290) (**Table 4**). The use of mTOR inhibitors in combination proved to be safe and was able to stabilize disease progression in some children with recurrent or refractory brain ependymoma (28, 29). Further phase II trials are ongoing to assess their anti-tumor activity (NCT02155920; NCT02574728) (**Table 2**). The EGFR inhibitor erlotinib was compared with etoposide in a phase II study in pediatric patients with recurrent ependymoma, but its efficacy was limited (114). A phase I trial is evaluating the safety profile and efficacy of HER2-targeting CAR-T cell therapy in recurrent and progressive ependymoma (NCT04903080) (**Table 4**). Similarly, a phase I study of the anti-PD-L1 pembrolizumab (**Figure 2**) in younger patients with recurrent and refractory HGG, ependymoma, and medulloblastoma is recruiting (NCT02359565) (**Table 3**). Other interesting targets for specific molecules include EZHIP, PARP, HDAC, the chemokine receptor CXCR4, the RAF/MEK/ERK pathway for NF2-associated tumors, and the Wnt-β-catenin (whose activation seems to be promoted by YAP1) and RELA pathways (since RELA mediates the activation of the NFkB pathway) (115).

### Embryonal tumors

Embryonal tumors account for 10-15% of primary CNS tumors in children and adolescents. Medulloblastoma (MB) is the most common, representing about 70% of embryonal tumors (92). The WHO 2021 classification distinguishes various molecular and histological subgroups. Among them, WNT-activated MB has a good long-term prognosis, since it is usually responsive to the currently available treatments (100). It harbors *CTNNB1* somatic mutations and chromosome 6 monosomy in almost all cases (80-90%) (116). *APC* pathogenic variants are generally identified in *CTNNB1* wild-type tumors, explaining the WNT pathway activation (117). Other recurrent mutations can affect the *DDX3X*, *SMARCA4*, *TP53*, *CSNK2B*, *PIK3CA*, and *EPHA7* genes (118). SHH-activated MB is more common in infants and adults and has an intermediate prognosis. The WHO 2021 classification distinguishes SHH-driven MB into *TP53*-mutant or wildtype. The activation of the SHH signaling pathway represents the most common genetic event, caused by mutations or deletions in *PTCH1* and *SUFU* genes, *SMO* activating mutations, *MYC*/*MYCN* or *GLI1*/*GLI2* amplifications. In addition, alterations in p53 and PI3K pathways can drive tumorigenesis. *MYC*/*MYCN* amplification and *TP53* mutations have been related to poor prognosis (119). Other frequent chromosomal alterations include the loss of chromosomes 9q (causing loss of heterozygosity of *PTCH1*), 10q, 14q, and 17p, and gains of chromosomes 2, 3q, and 9p (120, 121). A common driver pathway that defines group-3 and -4 MBs has not yet been identified. However, these subtypes share some genetic aberrations with the WNT and SHH subtypes. Distinctive features include *OTX2* and *CDK6* amplifications, *SMARC4*, *KBTBD4*, *CTDNEP1*, *KDM6A*, *ZMYM3* and *KTM2C* mutations, *PRDM6* overexpression (group 4), isochromosome 17q (present in about 50% of cases in both subgroups), loss of chromosomes 8, 10q, 11p, 16q and X and gain of chromosomes 1q, 7, and 18 (118, 121) (**Table 5**).

Standard treatment of MB consists of surgical tumor resection with craniospinal irradiation (except in infants) and chemotherapy, depending on risk stratification. The first-line chemotherapy consists of cisplatin, vincristine, and cyclophosphamide, while in MB metastatic at diagnosis, the combination of cyclophosphamide, vincristine, methotrexate, carboplatin, etoposide, and concomitant intraventricular methotrexate allowed achieving acceptable survival rates (122). The use of targeted approaches is rapidly evolving. Smoothened inhibitors (SMOi), such as vismodegib and sonidegib, have shown temporary activity in SHH-activated MB, but they have been associated with severe growth deceleration due to premature growth plate fusion, restricting their use to older adolescents and young adults (58, 59). Prexasertib, a CHK1/2 inhibitor, is being investigated in combination with chemotherapy in pediatric refractory or recurrent group 3, group 4, and SHH-activated MB (NCT04023669). A phase I/II trial to evaluate the CHK2 inhibitor silmitasertib in children with recurrent, progressive, or refractory SHH-activated MB is recruiting (NCT03904862) (**Table 4**).

ATRT is a rare CNS embryonal tumor that usually affects children in the first years of life. It has a poor prognosis since it usually grows fast and spreads through the cerebrospinal fluid (123). The main recurrent molecular aberration of ATRT is biallelic loss of function of SMARCB1, resulting from pathogenic variants, mutations, or partial or whole loss of chromosome 22. Rare cases (<5%) of SMARCB1-wildtype ATRT generally harbor SMARCA4 mutations (124) (**Table 5**). Based on DNA methylation profiling and gene expression, other recurrent molecular features have been identified and related to three ATRT subtypes, namely the overexpression of the SHH and Notch pathways (ATRT-SHH), the upregulation of the melanosomal pathway (ATRT-TYR), and the overexpression of the *MYC* oncogene and the *Hox* cluster (ATRT-MYC) (125).

The most common approach to ATRT is an aggressive multimodal treatment consisting of maximal safe surgical resection, followed by chemotherapy +/- radiotherapy. Considering its toxicity, various trials have been carried out with the goal of avoiding radiation therapy, especially in children <3 years (126). Standard chemotherapy is mainly based on two regimens, the first including etoposide, vincristine, cisplatin and cyclophosphamide, the second including etoposide, vincristine, carboplatin and ifosfamide. Additional high-dose chemotherapy (carboplatin + thiotepa + etoposide and cyclophosphamide + melphalan) followed by autologous HSCT is usually administered in high-risk patients (127). A phase I study of tazemetostat, a selective EZH2 inhibitor, in children with relapsed or refractory *SMARCB1*-negative tumors has provided promising interim results (NCT02601937) (**Table 2**). Another phase I/II trial of a combination regimen (*i.e.*, tazemetostat, the anti-PD-1 nivolumab, and the anti-CTLA-4 ipilimumab) in *SMARCB1* or *SMARCA4*-deficient neoplasms has just been designed (NCT05407441) (**Table 3**). The CDK4/6 inhibitor ribociclib was evaluated in combination with the mTOR inhibitor everolimus in children with recurrent, progressive or refractory brain cancers and was shown to be well tolerated (29), while a phase II trial of alisertib, an aurora A kinase inhibitor, is recruiting (NCT02114229) (**Table 4**). Furthermore, a phase I/II study will evaluate the efficacy of a combination regimen with the immune checkpoint inhibitors atezolizumab (anti-PD-L1) and tiragolumab (a novel anti-T-cell immunoreceptor with Ig and ITIM domains, TIGIT) in relapsed and refractory *SMARCB1* or *SMARCA4*-deficient tumors (NCT05286801) (**Table 3**).

Since recurrent CNS neoplasms are often genetically distinct from the primary one and resistant to treatments, a phase I trial is ongoing to evaluate rational combination therapies in refractory, relapsed, or recurrent brain tumors, based on tumor type and molecular characteristics (NCT03434262).

### Germ cell tumors

Germ cell tumors (GCTs) represent approximately 3% of pediatric primary CNS tumors worldwide but have higher incidence rates in East Asia, especially Japan and South Korea (92). They have traditionally been classified into germinoma, which accounts for 50-70% of cases, and non-germinomatous GCTs, which include many entities with a variable prognosis. The histopathological, molecular, and therapeutic features of intracranial GCTs are similar to the extracranial ones (128). The KIT/RAS and AKT/mTOR pathways are commonly involved. *KIT* mutations represent the most frequent molecular feature, followed by *KRAS*, *NRAS*, and *CBL* mutations, all resulting in KIT overexpression (129). Some studies reported that KIT/RAS pathway mutations were significantly more frequent in germinomas and in male patients (130). *AKT1* copy number gains were found in a high percentage of tumors with wildtype *KIT*, *KRAS*, and *NRAS* (129). Chromosomal instability is also common in intracranial GCTs. Gains of chromosomes 12p or X, and loss of 13q have been also seldom described and were found to significantly worsen prognosis (131) (**Table 5**).

The treatment of CNS GCTs combines the use of multiple chemotherapy agents, including carboplatin, etoposide, ifosfamide, and cyclophosphamide, and radiation therapy, while surgery plays a less established role, apart from non germinomatous tumors (132). So far, the field of targeted therapies has been poorly explored in GCTs. The TKI imatinib (**Figure 2**) was evaluated in children with recurrent or refractory CNS tumors expressing KIT and/or PDGFRA, proving to be safe but not particularly effective (133). A retrospective study analyzed the feasibility and tolerability of dasatinib (**Figure 2**), another TKI with improved CNS penetration, in patients with newly diagnosed or recurrent CNS germinoma (22), suggesting a potential role in future treatment strategies (**Table 2**).

### Other tumors

In recent years, the use of the term primitive neuroectodermal tumors (PNETs) has been questioned. Advanced molecular analyses revealed that most of PNETs can either be classified into other known CNS tumors (*e.g.*, HGG, ependymoma, embryonal tumors) or in new molecularly defined entities (134). In the 2021 WHO classification, two embryonal tumor subtypes were introduced: CNS neuroblastoma *FOXR2*-activated and CNS tumor with *BCOR* internal tandem duplication (100). The primary intracranial sarcoma *DICER1*-mutant and the *CIC*-rearranged sarcoma are now included in the mesenchymal tumors group. Another genetically defined new entity is CNS high-grade neuroepithelial tumor with *MN1* alteration (135) (**Table 5**). So far, no specific therapeutic protocols have been developed for these rare CNS tumor subtypes. The presence of distinct molecular features is attractive for the use of targeted drugs, but it requires further evaluation (134).

## Conclusions

In the last few decades, we have witnessed a rapid evolution of available options for treating childhood cancer, following the development of multiple molecules targeting specific mutations and pathomechanisms. Novel, molecular alterations have been identified, as well as the role of germline variants in childhood cancer development (up to 10-15% of cases). The identified molecular abnormalities resulted in multiple investigations of new targeted treatments. These drugs were often investigated in monotherapy, which probably limited their efficacy, and combination therapies should be rapidly introduced in clinical investigations. However, some of these agents have already proven useful as add-on therapy and are now included in the standard of care. Nevertheless, we have to acknowledge that many childhood solid cancers remain burdened by high mortality rates and severe *sequelae*. In the following years, our efforts should be oriented in multiple directions. On one hand, improving patients’ access to tumor profiling, in both high- and low-income countries, will guarantee a deeper understanding of the molecular landscape of childhood cancer. On the other hand, therapeutic efforts should be directed to the validation of available options within structured protocols and to the constant development of new molecules. Finally, a deeper cross-talk among clinicians by implementing multidisciplinary tumor boards (136), and between clinicians and caregivers, would certainly be beneficial.

## Author contributions

IB and FP performed the literature review and wrote the manuscript, while AT and CF critically revised it. All authors have read and approved the final version of the manuscript.
